# Cytokine–Cytokine Receptor Interaction and Endocytosis are Common Pathways for Symptom Burden and Sickness Behavior Symptoms in Oncology Patients Undergoing Chemotherapy

**DOI:** 10.1002/cam4.71328

**Published:** 2025-11-11

**Authors:** Carolyn S. Harris, Kord M. Kober, Joosun Shin, Lisa Morse, Kate R. Oppegaard, Steven Paul, Marilyn J. Hammer, Jon D. Levine, Yvette P. Conley, Christine A. Miaskowski

**Affiliations:** ^1^ School of Nursing, University of California San Francisco California USA; ^2^ School of Nusing, University of California Los Angeles California USA; ^3^ VA Portland Health Care System Portland Oregon USA; ^4^ Dana‐Farber Cancer Institute Boston Massachusetts USA; ^5^ School of Medicine, University of California San Francisco California USA; ^6^ School of Nursing, University of Pittsburgh Pittsburgh Pennsylvania USA

**Keywords:** cancer, chemotherapy, cutpoints, gene expression, immune system, inflammation

## Abstract

**Background:**

Inflammation is associated with sickness behavior symptoms in patients receiving chemotherapy. However, its impact on symptom burden (i.e., higher number of concurrent symptoms) requires evaluation. Study purposes were to evaluate for differentially perturbed immune and/or inflammatory pathways between outpatients receiving chemotherapy with Low (i.e., 0–8) versus High (i.e., 16–38) symptom burden and identify common immune and/or inflammatory pathways among symptom burden and single sickness behavior symptoms.

**Methods:**

Prior to their second or third cycle of chemotherapy, oncology outpatients reported the occurrence of 38 symptoms using the Memorial Symptom Assessment Scale and provided a peripheral blood sample. Using previously identified symptom cutpoints, patients with Low versus High symptom burden were evaluated. Transcriptome‐wide gene expression was quantified using RNA‐sequencing (*n* = 213; RNA‐seq sample) or microarray (*n* = 207; microarray sample) technologies. Pathway impact analyses (PIA) were performed and signaling pathways were defined with the Kyoto Encyclopedia of Genes and Genomes database. Fisher's combined probability test was used to identify perturbed pathways between the Low and High symptom burden groups across both samples (false discovery rate < 0.005).

**Results:**

For the RNA‐seq sample, 159 patients had High and 54 patients had Low symptom burden. For the microarray sample, 135 patients had High and 72 patients had Low symptom burden. Of the 40 pathways that were perturbed between the Low and High symptom burden groups, 10 were involved in immune or inflammatory processes. Cytokine–cytokine receptor interaction and endocytosis pathways were the common pathways identified across this study and PIAs of single sickness behavior symptoms.

**Conclusions:**

This study is the first to identify differentially perturbed immune and inflammatory signaling pathways that were associated with symptom burden in oncology patients receiving chemotherapy. Evaluation of interventions targeted at the cytokine–cytokine receptor interaction and endocytosis pathways may decrease the burden associated with single and multiple occurring symptoms.

## 1 Introduction

Despite advances in treatment, patients with cancer experience a substantial symptom burden. Symptom burden is conceptualized as the subjective report of multiple concurrent symptoms that cumulatively “may produce multiple negative physical, psychological, and emotional patient responses” (p. 677) [1]. Among patients receiving cancer treatment, a higher symptom burden is associated with poorer performance status [2, 3] and decrements in quality of life [2, 4]. In addition, a higher symptom burden may lead to dose reductions and/or delays or even cessation of treatments that can decrease survival [5].

Symptom burden is often operationalized as a mean or composite score. However, these methods do not account for interindividual variability in patients' symptom experiences. For example, in one study of patients receiving chemotherapy [6], while the mean number of symptoms for the total sample was 13.9 (±7.2), patients reported zero to 38 co‐occurring symptoms. The creation of clinically meaningful cutpoints is one method to account for this variability by categorizing symptom scores into discrete categories. Previously applied to pain [7] and fatigue [8], clinically meaningful cutpoints are essential clinical tools that facilitate the identification of patients who require an intervention.

In a study by our team [9], clinically meaningful cutpoints for symptom burden were identified in a large sample of patients receiving chemotherapy (*n* = 1329). Using occurrence rates for 38 symptoms commonly associated with cancer and its treatment from a modified version of the Memorial Symptom Assessment Scale (MSAS) [10], the optimal cutpoints for Low (i.e., 0–8 symptoms), Moderate (i.e., 9–15 symptoms), and High (i.e., 16–38 symptoms) symptom burden were determined. Of note, 38.4% of the patients were in the High group. The symptoms with the highest occurrence rates in this group were: lack of energy, difficulty sleeping, feeling drowsy, pain, worrying, difficulty concentrating, and nausea.

Of note, the majority of symptoms listed in the previous sentence are characteristic of “sickness behavior” that is observed following an inflammatory insult (e.g., lipopolysaccharide, chemotherapy) [11, 12, 13]. In patients with cancer, it is hypothesized that sickness behavior develops as a result of immune system activation by cancer, cancer treatments, and/or stress [14, 15, 16]. Specifically, proinflammatory cytokines that are released in the periphery transmit inflammatory signals to the brain via afferent nerves and cross the blood‐brain barrier [11]. There, these proinflammatory cytokines activate neurological systems and stimulate the production of additional proinflammatory cytokines within the brain.

While no study has evaluated the molecular mechanisms associated with a higher symptom burden, several preclinical studies have evaluated associations between chemotherapy‐induced sickness behavior and differential expression of inflammatory genes [17, 18, 19, 20, 21]. In one study [17], compared to saline, female mice treated with cyclophosphamide, doxorubicin, and 5‐fluorouracil demonstrated decreases in voluntary wheel running activity, food intake, and body weight. In addition, the chemotherapy‐treated mice had increased expression of interleukin 1 beta (*Il1b*) in the serum, liver, spleen, and cerebellum and tumor necrosis factor alpha (*Tnf*) in the cerebellum.

Across three studies that evaluated associations between paclitaxel‐induced sickness behavior and differential expression of several inflammatory genes (i.e., *Il1b*, *Il6*, *Il10*, *Tnf*, C‐X‐C motif chemokine ligand 1 (*Cxcl1*), intercellular adhesion molecule 1) in female mice [18, 20, 21], compared to vehicle, paclitaxel‐treated mice had lower body mass and displayed decreases in food intake, voluntary wheel running, home locomotion, and/or open field central tendency (i.e., tendency to avoid central areas of an open field, measurement of anxiety‐like behavior). However, plasma and gene expression levels of inflammatory markers across various brain regions were not consistent across these studies.

In another study that used a syngeneic heterotopic murine model of human papilloma virus‐related head and neck cancer treated with cisplatin and radiation therapy [19], compared to healthy male mice, the treated mice displayed decreased body weight and burrowing. However, none of the inflammatory genes that were evaluated (i.e., *Il6*, *Il1b*, *Tnf*) were differentially expressed in the tumor, liver, or whole brain.

While these preclinical findings support an association between sickness behavior and inflammatory markers in animals treated with various types of chemotherapy, findings were not consistent. These inconsistent findings may be related to differences in the animals' sex, types of chemotherapy administered, type of tissue evaluated (e.g., tumor, liver), and sample sizes (i.e., 3–20 mice per comparison group). In addition, while a targeted evaluation of inflammatory markers can be informative, this approach does not account for the inherent complexities of biological processes (e.g., gene–gene and gene–gene product interactions).

Pathway analysis is one approach that, through the use of differential gene expression data, accounts for these complex interactions and identifies perturbed (i.e., impacted) biological signaling pathways [22]. To conduct these types of analyses, a two‐group comparison is a common method that is employed. Comparison of groups of patients at the extremes of a complex phenotype (e.g., low versus high symptom burden) is one successful approach to strengthen the biological signal and identify biological processes underlying that phenotype [23]. As compared to a two‐group comparison of healthy controls and patients with cancer, an extreme phenotype approach makes sense clinically because it will help to identify biological characteristics that distinguish oncology patients with low and high symptom burden.

In previous work by our team that evaluated associations between individual sickness behavior symptoms (i.e., anxiety [24], cognitive impairment [25], morning and evening fatigue [26], nausea [27, 28], pain [29], sleep disturbance [30], depression [unpublished], shortness of breath [31]) and differentially perturbed pathways in patients receiving chemotherapy, several common immune and inflammatory pathways were identified. Given that in our cutpoint analysis [9], these symptoms had the highest occurrence rates in the High symptom burden group (e.g., difficulty sleeping = 87.7%, pain = 82.8%), it is plausible that one or more of these immune and/or inflammatory pathways contribute to a higher symptom burden through additive or synergistic effects. An increased understanding of these processes will inform the development and testing of interventions to decrease symptom burden.

Therefore, building on our previous work that identified an optimal set of cutpoints for symptom burden [9], the purpose of this study was to evaluate differentially perturbed immune and inflammatory pathways between outpatients receiving chemotherapy with a Low (i.e., 0–8) versus a High (i.e., 16–38) symptom burden. Then, building on our previous work that identified associations between several immune and/or inflammatory pathways and single symptoms [24, 25, 26, 27, 28, 29, 30, 31], common immune and/or inflammatory pathways were identified.

## 2 Methods

### 2.1 Patients and Settings

This study is part of a larger, longitudinal study of the symptom experience of oncology outpatients receiving chemotherapy [32]. Eligible patients were ≥ 18 years of age; had a diagnosis of breast, gastrointestinal, gynecological, or lung cancer; had received chemotherapy within the preceding four weeks; were scheduled to receive at least two additional cycles of chemotherapy; were able to read, write, and understand English; and gave written informed consent. Patients were recruited from two Comprehensive Cancer Centers, one Veterans Affairs hospital, and four community‐based oncology programs.

### 2.2 Study Procedures

This study was approved by the Institutional Review Board at each of the study sites. Of the 2234 patients approached, 1343 consented to participate (60.1% response rate). The major reason for refusal was being overwhelmed with their cancer treatment. Eligible patients were approached in the infusion unit during their first or second cycle of chemotherapy by a member of the research team to discuss study participation and obtain written informed consent.

Demographic, clinical, symptom, and molecular data were collected at enrollment (i.e., the week prior to the patient's second or third cycle of chemotherapy). As part of the patients' routine pre‐chemotherapy evaluation, whole blood was collected into PAXgene ribonucleic acid (RNA) stabilization tubes. Total RNA was isolated according to the manufacturer's standard protocol (Qiagen, USA) using the PaxGene Blood RNA Kit. All of the samples retained for gene expression profiling demonstrated an RNA integrity number of ≥ 8.

Because techniques for quantification of gene expression changed over time, of the 717 patients who provided a blood sample for the parent study, 357 patients had their samples processed using RNA sequencing (i.e., RNA‐seq sample; Figure S1) and 360 patients had their samples processed using microarray (i.e., microarray sample) technologies. The current analysis used the gene expression data from the patients in the extreme symptom burden groups (i.e., Low, High) that were obtained using the RNA‐seq (*n* = 213) and microarray (*n* = 207) technologies.

### 2.3 Instruments

#### 2.3.1 Demographic and Clinical Characteristics

Patients completed a demographic questionnaire, Karnofsky Performance Status (KPS) scale [33], Self‐Administered Comorbidity Questionnaire (SCQ) [34], and Alcohol Use Disorders Identification Test [35]. The toxicity of each patient's chemotherapy regimen was rated using the MAX2 index [36]. Medical records were reviewed for disease and treatment information.

#### 2.3.2 Memorial Symptom Assessment Scale

A modified version of the MSAS was used to evaluate the occurrence of 38 symptoms commonly associated with cancer and its treatment [10]. Based on a review of the literature and evaluation of other multidimensional symptom scales published after the MSAS, the following six symptoms were added to the original 32 MSAS symptoms: hot flashes, chest tightness, difficulty breathing, abdominal cramps, increased appetite, and weight gain. Using the MSAS, patients were asked to indicate whether or not they had experienced each symptom in the past week (i.e., symptom occurrence). The reliability and validity of the MSAS are well established in studies of inpatients and outpatients with cancer [10].

### 2.4 Data Analysis

#### 2.4.1 Determination of Symptom Cutpoints

Symptom cutpoints were determined as previously reported using data from the enrollment assessment [9]. In brief, multiple cutpoints for the occurrence of 38 common cancer‐related symptoms were evaluated against the Quality of Life Scale‐Patient Version mean total scores using analysis of variance (ANOVA). Optimal cutpoints for low, moderate, and high symptom burden were determined by identifying the symptom occurrence threshold (i.e., ANOVA that yielded the largest F ratio for the between‐category effects) associated with significant differences in quality of life. The optimal cutpoints were 0–8 symptoms for Low, 9–15 symptoms for Moderate, and 16–38 symptoms for High symptom burden groups [9]. Measures of global [37, 38], disease‐specific [39, 40], and cumulative life stress [41] were used to validate these cutpoints. The current study evaluated patients in the Low versus the High symptom burden groups.

#### 2.4.2 Identification of Demographic and Clinical Characteristics for Use in the Logistic Regression Analyses

Patient demographic and clinical data from the RNA‐seq and microarray samples were analyzed separately using IBM SPSS Statistics Version 27 (IBM Corporation, Armonk, NY). Differences in demographic and clinical characteristics between the Low and High symptom burden groups were evaluated using parametric and nonparametric tests. Significant characteristics (*p* < 0.05) were used as covariates in the logistic regression analyses.

Separate regression models were done for the RNA‐seq and microarray samples. The dependent variable for each regression model was membership in the High symptom burden group. Using a backward stepwise approach, logistic regression analyses were used to determine significant demographic and clinical covariates for inclusion in the differential expression analyses.

#### 2.4.3 Differential Gene Expression Analyses

Details on the gene expression methods and pathway impact analyses are described elsewhere [27]. In brief, differential expression was quantified using empirical Bayes models that were implemented using edgeR [42] for the RNA‐seq sample and limma [43] for the microarray sample. These analyses included demographic and clinical covariates that were retained in the final logistic regression models. In addition, the models included surrogate variables which were identified using surrogate variable analysis that adjusted for variation due to unmeasured sources [44]. Expression loci were annotated with Entrez gene identifiers. Gene symbols were derived and matched using the HUGO Gene Nomenclature Committee resource database [45]. The differential expression results were summarized as the log fold‐change and *p*‐value for each gene. Only genes that had a common direction of expression (i.e., log‐fold change) across the two samples were retained for subsequent analyses.

#### 2.4.4 Pathway Impact Analysis (PIA)

The PIAs included potentially important biological factors (e.g., gene–gene interactions, flow signals in a pathway) as well as the magnitude (i.e., log fold change) and *p*‐values from the differential expression analysis for each sample [22]. The PIAs included the results of the differential expression analysis for all genes (i.e., cutoff free) that had a common direction of differential expression to determine the probability of pathway perturbations using Pathway Express (version 2.18.0) [46]. For each sample (i.e., RNA‐seq, microarray), a separate test was performed for each pathway. A total of 221 signaling pathways were defined using the Kyoto Encyclopedia of Genes and Genomes (KEGG) database [47].

#### 2.4.5 Meta‐Analysis of the PIAs

To improve statistical power [48], a meta‐analysis approach was used to combine the final results from the RNA‐seq and microarray PIAs. Fisher's combined probability method was used to combine the test results from both samples to obtain a single test (global) of the null hypothesis. The significance of the combined transcriptome‐wide PIA was assessed using a false discovery rate (FDR) of < 0.005 under the Benjamini–Hochberg procedure [49]. Then, the significantly perturbed pathways were evaluated for immune and/or inflammatory processes. Finally, common immune or inflammatory pathways across this study and our previous studies [24, 25, 26, 27, 28, 29, 30, 31] of single symptoms were identified.

## 3 Results

### 3.1 Differences in Demographic and Clinical Characteristics

Of the 214 patients in the RNA‐seq sample with complete phenotypic data, 25.7% were in the Low and 74.3% were in the High symptom burden groups (Table 1). Compared to the Low group, the High group was younger and more likely to be female. In addition, the High group had lower KPS scores, a higher number of comorbid conditions, higher comorbidity burden, was more likely to self‐report a diagnosis of depression or back pain, and was less likely to have gastrointestinal cancer or high blood pressure.

**TABLE 1:** Differences in demographic and clinical characteristics between patients with low and high symptom burden in the RNA‐Seq sample.

Characteristic	Low symptom group (0–8), 25.7% (*n* = 55)	High symptom group (16–38), 74.3% (*n* = 159)	Statistics
	Mean (SD)	Mean (SD)	
Age (years)	60.5 (13.5)	54.1 (12.6)	*t* = 3.17, *p* = 0.002
Education (years)	16.3 (3.0)	15.5 (3.0)	*t* = 1.67, *p* = 0.096
Body mass index (kg/m^2^)	25.5 (3.9)	25.9 (6.3)	*t* = −0.67, *p* = 0.507
Karnofsky Performance Status score	87.1 (11.2)	73.8 (11.6)	*t* = 7.43, *p* < 0.001
Number of comorbid conditions	2.2 (1.4)	2.7 (1.6)	*t* = −2.00, *p* = 0.046
Self‐administered Comorbidity Questionnaire score	4.8 (3.0)	6.4 (3.8)	*t* = −3.30, *p* = 0.001
Alcohol Use Disorders Identification Test score	3.0 (1.8)	2.9 (2.7)	*t* = 0.32, *p* = 0.746
Time since diagnosis (years)	1.4 (2.3)	1.5 (2.7)	*U*, *p* = 0.732
Time since diagnosis (years, median)	0.45	0.43	
Number of prior cancer treatments	1.5 (1.3)	1.4 (1.4)	*t* = 0.19, *p* = 0.850
Number of metastatic sites including lymph node involvement^a^	1.3 (1.0)	1.2 (1.3)	*t* = 0.66, *p* = 0.509
Number of metastatic sites excluding lymph node involvement	0.69 (0.88)	0.79 (1.1)	*t* = −0.65, *p* = 0.519
MAX2 score	0.17 (0.08)	0.19 (0.08)	*t* = −1.73, *p* = 0.085
	**% (*n*)**	**% (*n*)**	
Gender			FE, *p* < 0.001
Female	61.8 (34)	86.8 (138)	
Male	38.2 (21)	13.2 (21)	
Self‐reported race or ethnicity			*Χ* ^2^ = 1.57, *p* = 0.666
Asian or Pacific Islander	14.8 (8)	13.9 (22)	
Black	5.6 (3)	7.0 (11)	
Hispanic, Mixed, or Other	7.4 (4)	13.3 (21)	
White	72.2 (39)	65.8 (104)	
Married or partnered (% yes)	58.2 (32)	59.2 (93)	FE, *p* = 1.000
Lives alone (% yes)	23.6 (13)	23.4 (37)	FE, *p* = 1.000
Currently employed (% yes)	35.2 (19)	27.8 (44)	FE, *p* = 0.307
Annual household income			*U*, *p* = 0.057
Less than $30,000	13.0 (6)	26.7 (39)	
$30,000 to $70,000	17.4 (8)	20.5 (30)	
$70,000 to $100,000	21.7 (10)	15.1 (22)	
Greater than $100,000	47.8 (22)	37.7 (55)	
Childcare responsibilities (% yes)	21.8 (12)	26.4 (42)	FE, *p* = 0.599
Elder care responsibilities (% yes)	0.0 (0)	10.1 (16)	n/a
Past or current history of smoking (% yes)	36.4 (20)	38.2 (60)	FE, *p* = 0.872
Exercise on a regular basis (% yes)	68.5 (37)	65.8 (100)	FE, *p* = 0.741
Specific comorbid conditions (% yes)			
Heart disease	5.5 (3)	7.5 (12)	FE, *p* = 0.765
High blood pressure	41.8 (23)	27.0 (43)	FE, *p* = 0.044
Lung disease	5.5 (3)	11.9 (19)	FE, *p* = 0.207
Diabetes	9.1 (5)	8.2 (13)	FE, *p* = 0.784
Ulcer or stomach disease	3.6 (2)	5.0 (8)	FE, *p* = 1.000
Kidney disease	1.8 (1)	0.6 (1)	FE, *p* = 0.449
Liver disease	3.6 (2)	6.9 (11)	FE, *p* = 0.522
Anemia or blood disease	7.3 (4)	12.6 (20)	FE, *p* = 0.333
Depression	9.1 (5)	30.8 (49)	FE, *p* = 0.001
Osteoarthritis	12.7 (7)	13.2 (21)	FE, *p* = 1.000
Back pain	18.2 (10)	42.8 (68)	FE, *p* = 0.001
Rheumatoid arthritis	7.3 (4)	6.3 (10)	FE, *p* = 0.759
Cancer diagnosis			*Χ* ^2^ = 8.58, *p* = 0.035
Breast cancer	30.9 (17)	43.4 (69)	NS
Gastrointestinal cancer	49.1 (27)	27.7 (44)	0 > 1
Gynecological cancer	10.9 (6)	17.6 (28)	NS
Lung cancer	9.1 (5)	11.3 (18)	NS
Prior cancer treatment			*Χ* ^2^ = 3.74, *p* = 0.291
No prior treatment	25.0 (13)	31.2 (48)	
Only surgery, CTX, or RT	38.5 (20)	42.9 (66)	
Surgery and CTX, or surgery and RT, or CTX and RT	25.0 (13)	13.6 (21)	
Surgery and CTX and RT	11.5 (6)	12.3 (19)	
Cycle length			*U*, *p* = 0.580
14‐day cycle	49.1 (27)	47.2 (75)	
21‐day cycle	47.3 (26)	44.0 (70)	
28‐day cycle	3.6 (2)	8.8 (14)	
Emetogenicity of the CTX regimen			*U*, *p* = 0.945
Minimal/low	10.9 (6)	18.2 (29)	
Moderate	72.7 (40)	59.1 (94)	
High	16.4 (9)	22.6 (36)	
Antiemetic regimen			*Χ* ^2^ = 6.98, *p* = 0.072
None	5.5 (3)	3.8 (6)	
Steroid alone or serotonin receptor antagonist alone	23.6 (13)	17.8 (28)	
Serotonin receptor antagonist and steroid	56.4 (31)	45.2 (71)	
NK‐1 receptor antagonist and two other antiemetics	14.5 (8)	33.1 (52)	

Abbreviations: CTX, chemotherapy; FE, Fisher's exact test; kg, kilograms; m^2^, meters squared; n/a, not applicable; NK‐1, neurokinin‐1; NS, not significant; RNA, ribonucleic acid; RT, radiation therapy; SD, standard deviation; *U*, Mann–Whitney *U* test.

^a^ Total number of metastatic sites evaluated was 9.

Of the 207 patients in the microarray sample, 34.8% were in the Low group and 65.2% were in the High group (Table 2). Compared to the Low group, the High group was younger, more likely to be female, more likely to have a lower annual income, and less likely to be married or partnered. In addition, the High group had lower KPS scores, a higher body mass index, a higher number of comorbid conditions, a higher comorbidity burden, and was more likely to self‐report a diagnosis of anemia, depression, or back pain.

**TABLE 2:** Differences in demographic and clinical characteristics between patients with low and high symptom burden in the microarray sample.

Characteristic	Low symptom group (0–8), 34.8% (*n* = 72)	High symptom group (16–38), 65.2% (*n* = 135)	Statistics
	Mean (SD)	Mean (SD)	
Age (years)	59.7 (10.7)	54.6 (10.9)	*t* = 3.20, *p* = 0.002
Education (years)	16.5 (2.9)	15.8 (2.5)	*t* = 1.85, *p* = 0.066
Body mass index (kg/m^2^)	25.6 (5.7)	27.9 (6.6)	*t* = −2.40, *p* = 0.017
Karnofsky Performance Status score	83.9 (9.9)	76.1 (11.3)	*t* = 4.94, *p* < 0.001
Number of comorbid conditions	2.0 (1.2)	2.8 (1.4)	*t* = −4.37, *p* < 0.001
Self‐administered Comorbidity Questionnaire score	4.5 (2.5)	6.6 (3.1)	*t* = −5.23, *p* < 0.001
Alcohol Use Disorders Identification Test score	3.2 (2.7)	3.1 (3.1)	*t* = 0.11, *p* = 0.913
Time since diagnosis (years)	1.9 (3.5)	2.5 (3.9)	*U*, *p* = 0.130
Time since diagnosis (years, median)	0.45	0.43	
Number of prior cancer treatments	1.8 (1.7)	1.9 (1.6)	*t* = −0.44, *p* = 0.659
Number of metastatic sites including lymph node involvement^a^	1.4 (1.4)	1.2 (1.3)	*t* = 0.77, *p* = 0.444
Number of metastatic sites excluding lymph node involvement	0.94 (1.3)	0.80 (1.1)	*t* = 0.85, *p* = 0.399
MAX2 score	0.16 (0.09)	0.18 (0.08)	*t* = −1.26, *p* = 0.208
	**% (*n*)**	**% (*n*)**	
Gender			
Female	73.6 (53)	88.9 (120)	FE, *p* = 0.006
Male	26.4 (19)	11.1 (15)	
Self‐reported race or ethnicity			
Asian or Pacific Islander	12.9 (9)	12.0 (16)	*Χ* ^2^ = 387, *p* = 0.275
Black	1.4 (1)	8.3 (11)	
Hispanic, Mixed, or Other	10.0 (7)	9.8 (13)	
White	75.7 (53)	69.9 (93)	
Married or partnered (% yes)	75.0 (54)	57.8 (78)	FE, *p* = 0.015
Lives alone (% yes)	16.9 (12)	22.2 (30)	FE, *p* = 0.467
Currently employed (% yes)	38.9 (28)	25.9 (35)	FE, *p* = 0.059
Annual household income			
Less than $30,000	15.3 (11)	25.9 (35)	*U*, *p* < 0.001
$30,000 to $70,000	12.5 (9)	26.7 (36)	
$70,000 to $100,000	16.7 (12)	14.8 (20)	
Greater than $100,000	55.6 (40)	32.6 (44)	
Childcare responsibilities (% yes)	19.4 (14)	27.1 (36)	FE, *p* = 0.239
Elder care responsibilities (% yes)	6.1 (4)	10.7 (13)	FE, *p* = 0.426
Past or current history of smoking (% yes)	34.7 (25)	39.1 (52)	FE, *p* = 0.550
Exercise on a regular basis (% yes)	73.2 (52)	67.9 (91)	FE, *p* = 0.523
Specific comorbid conditions (% yes)			
Heart disease	8.3 (6)	4.4 (6)	FE, *p* = 0.349
High blood pressure	26.4 (19)	30.4 (41)	FE, *p* = 0.630
Lung disease	11.1 (8)	9.6 (13)	FE, *p* = 0.810
Diabetes	5.6 (4)	10.4 (14)	FE, *p* = 0.306
Ulcer or stomach disease	0.0 (0)	7.4 (10)	n/a
Kidney disease	0.0 (0)	1.5 (2)	n/a
Liver disease	9.7 (7)	7.4 (10)	FE, *p* = 0.600
Anemia or blood disease	5.6 (4)	20.7 (28)	FE, *p* = 0.004
Depression	8.3 (6)	36.3 (49)	FE, *p* < 0.001
Osteoarthritis	11.1 (8)	18.5 (25)	FE, *p* = 0.231
Back pain	15.3 (11)	33.3 (45)	FE, *p* = 0.005
Rheumatoid arthritis	1.4 (1)	3.7 (5)	FE, *p* = 0.667
Cancer diagnosis			
Breast cancer	36.1 (26)	42.2 (57)	*Χ* ^2^ = 7.66, *p* = 0.053
Gastrointestinal cancer	22.2 (16)	26.7 (36)	
Gynecological cancer	23.6 (17)	25.2 (34)	
Lung cancer	18.1 (13)	5.9 (8)	
Prior cancer treatment			
No prior treatment	22.2 (16)	16.4 (22)	*Χ* ^2^ = 1.20, *p* = 0.753
Only surgery, CTX, or RT	37.5 (27)	42.5 (57)	
Surgery and CTX, or surgery and RT, or CTX and RT	22.2 (16)	21.6 (29)	
Surgery and CTX and RT	18.1 (13)	19.4 (26)	
Cycle length			
14‐day cycle	29.2 (21)	35.6 (48)	*U*, *p* = 0.301
21‐day cycle	62.5 (45)	58.5 (79)	
28‐day cycle	8.3 (6)	5.9 (8)	
Emetogenicity of the CTX regimen			
Minimal/low	20.8 (15)	21.5 (29)	*U*, *p* = 0.783
Moderate	63.9 (46)	60 (81)	
High	15.3 (11)	18.5 (25)	
Antiemetic regimen			
None	15.5 (11)	6.1 (8)	*Χ* ^2^ = 7.45, *p* = 0.059
Steroid alone or serotonin receptor antagonist alone	26.8 (19)	21.4 (28)	
Serotonin receptor antagonist and steroid	43.7 (31)	48.1 (63)	
NK‐1 receptor antagonist and two other antiemetics	14.1 (10)	24.4 (32)	

Abbreviations: CTX, chemotherapy; FE, Fisher's exact test; kg, kilograms; m^2^, meters squared; n/a, not applicable; NK‐1, neurokinin‐1; RT, radiation therapy; SD, standard deviation; *U*, Mann–Whitney *U* test.

^a^ Total number of metastatic sites evaluated was 9.

### 3.2 Demographic and Clinical Characteristics Included in the Gene Expression Analyses

For the RNA‐seq sample (Table 3), four variables were retained in the final logistic regression model and were used as covariates in the gene expression analysis (i.e., gender, KPS score, SCQ score, self‐reported diagnosis of high blood pressure). For the microarray sample, four variables were retained in the final logistic regression model and were used as covariates in the gene expression analysis (i.e., age, KPS score, SCQ score, self‐reported diagnosis of depression).

**TABLE 3:** Multiple logistic regression analyses predicting high symptom burden group membership.

Predictors	Odds ratio	95% CI	*p*
RNA‐sequencing sample (*n* = 214)			
Karnofsky Performance Status score	0.91	0.88, 0.95	< 0.001
Self‐Administered Comorbidity Questionnaire score	1.23	1.04, 1.45	0.014
Female gender	3.35	1.43, 7.87	0.005
Self‐reported diagnosis of high blood pressure	0.28	0.11, 0.76	0.012
Overall model fit: df = 4, *X* ^2^ = 69.43, *p* < 0.001			
Microarray sample (*n* = 207)			
Age	0.95	0.91, 0.98	0.002
Karnofsky Performance Status score	0.95	0.92, 0.98	0.004
Self‐Administered Comorbidity Questionnaire score	1.30	1.13, 1.50	< 0.001
Self‐reported diagnosis of depression	3.41	1.27, 9.17	0.015
Overall model fit: df = 4, *X* ^2^ = 60.31, *p* < 0.001			

Abbreviations: CI, confidence interval; df, degrees of freedom.

### 3.3 Differential Gene Expression Analyses

For the RNA‐seq sample, the median library threshold size was 9,273,000 reads. Following the application of quality control filters, 13,301 genes were included in the final analysis. The common dispersion was estimated as 0.179, yielding a biological coefficient of variation of 0.423.

For the microarray sample, all of the samples demonstrated good hybridization performance for biotin, background negative, and positive control assays on the arrays. Limma was used for background correction, quantile normalization, and log2 transformation [43]. Following quality control filters, 44,589 probes were included in the final analysis.

### 3.4 PIA

For the RNA‐seq sample, 11 surrogate variables were identified and included in the final differential expression model. For the microarray sample, 18 surrogate variables were identified and included in the final differential expression model. For both samples, a total of 4261 genes were included in the PIAs. Across the two samples, 40 KEGG signaling pathways were significantly perturbed (Table S1). Ten of these pathways were related to immune or inflammatory processes (Table 4).

**TABLE 4:** Perturbed immune or inflammatory pathways between patients with low and high symptom burden.

Pathway ID	Pathway name	Combined analysis statistics
hsa04672	Intestinal immune network for immunoglobulin A production	*Χ* ^2^ = 30.41, *p* = 8.13 × 10^−5^
hsa04659	Helper T 17 cell differentiation	*Χ* ^2^ = 29.02, *p* = 1.14 × 10^−4^
hsa04621	Nucleotide‐binding oligomerization domain‐like receptor signaling pathway	*Χ* ^2^ = 23.74, *p* = 8.15 × 10^−4^
hsa04151	Phosphoinositide 3‐kinase protein kinase B signaling pathway	*Χ* ^2^ = 19.04, *p* = 4.38 × 10^−3^
hsa04064	Nuclear factor kappa B signaling pathway	*Χ* ^2^ = 19.44, *p* = 3.84 × 10^−3^
hsa04010	Mitogen‐activated protein kinase signaling pathway	*Χ* ^2^ = 23.67, *p* = 8.15 × 10^−4^
hsa04612	Antigen processing and presentation	*Χ* ^2^ = 30.41, *p* = 8.13 × 10^−5^
hsa04144	Endocytosis	*Χ* ^2^ = 23.74, *p* = 8.15 × 10^−4^
hsa04145	Phagosome	*Χ* ^2^ = 30.41, *p* = 8.13 × 10^−5^
hsa04060	Cytokine–cytokine receptor interaction	*Χ* ^2^ = 19.95, *p* = 3.31 × 10^−3^

*Note:* The global perturbation *p*‐value was adjusted with the Benjamini–Hochberg procedure.

Abbreviations: hsa, *Homo sapiens* ; ID, identifier.

## 4 Discussion

This study is the first to identify differentially perturbed signaling pathways between oncology patients with low and high symptom burden receiving chemotherapy. Consistent with our a priori hypothesis, ten of the perturbed pathways were involved in immune or inflammatory processes (Table 4). The first part of the Discussion describes these pathways and current evidence regarding their association with symptom burden. The Discussion concludes with a summary of the common pathways that were identified in the current analysis and our previous analyses of single sickness behavior symptoms (Table 5) [24, 25, 26, 27, 28, 29, 30, 31].

**TABLE 5:** Common perturbed immune or inflammatory pathways across symptom burden and single sickness behavior symptoms.

Symptom	Significance threshold	KEGG pathways									
		Cytokine–cytokine receptor interaction	Endocytosis	Antigen processing and presentation	Phagosome	NOD‐like receptor signaling pathway	Intestinal immune network for IgA production	Mitogen‐activated protein kinase signaling pathway	Th17 cell differentiation	PI3K‐Akt signaling pathway	NF‐kappa B signaling pathway
Symptom burden	FDR < 0.005	■	■	■	■	■	■	■	■	■	■
Shortness of breath^a^	FDR < 0.025	■	■	■	■	■	■	■	■	■	■
Morning fatigue^b^	FDR < 0.025	■	■	■	■	■	■	■	■		■
Anxiety^c^	FDR < 0.01	■	■	■	■		■	■	■	■	
Evening fatigue^b^	FDR < 0.025	■	■	■	■	■	■		■	■	
Sleep disturbance^d^	FDR < 0.015	■	■	■	■	■	■		■		
Cognitive impairment^e^	FWER < 0.05	■	*	*	*	*		■			
Depression	FDR < 0.01	*	*	*	*	*				*	
Nausea^f^, ^g^	FWER < 0.01	■	■	■			■	■		■	■
Pain^h^	FDR < 0.015	■	■		■	■		■	■		
Common pathways across symptoms		10/10	10/10	9/10	9/10	8/10	7/10	7/10	7/10	6/10	4/10

*Note:* * indicates unpublished findings.

Abbreviations: Akt, protein kinase B; FDR, false discovery rate; FWER, family‐wise error rate; IgA, immunoglobulin A; KEGG, Kyoto Encyclopedia of Genes and Genomes; NF‐kappa B, nuclear factor kappa‐light‐chain‐enhancer of activated B cells; NOD‐like, nucleotide‐binding oligomerization domain‐like; PI3K, phosphoinositide 3‐kinases; Th17, Helper T 17. The black square boxes indicate that the KEGG pathway (column) was identified for that symptom (row).

^a^ Shin et al. [31].

^b^ Kober et al. [26].

^c^ Oppegaard et al. [24].

^d^ Calvo‐Schimmel et al. [30].

^e^ Oppegaard et al. [25].

^f^ Singh et al. [27].

^g^ Singh et al. [28].

^h^ Shin et al. [29].

### 4.1 Intestinal Immune Network for Immunoglobulin A (IgA) Production

The intestinal mucosa consists of a network of immune cells (e.g., dendritic cells, B cells, T cells) that produce IgA and secretory IgA. Both forms of IgA play vital roles in passive immunity by regulating the microbiome and eliminating bacterial and viral pathogens that pose a threat to the intestinal mucosa [50]. In addition, IgA engages in active immunity by regulating cytokine production (e.g., TNF, IL‐1B) [50].

The administration of chemotherapy disrupts this network by altering the composition of the gastrointestinal microbiota; damaging the epithelial barrier; and impairing the intestinal immune response [51]. These disruptions lead to increases in pathogenic intestinal microbiota and translocation of toxins and pathogens from the gut into the systemic circulation [52] which increases local and central inflammation [21, 52]. These processes result in higher symptom burden through activation of the gut‐brain axis [52, 53].

A bidirectional communication channel connecting the gastrointestinal microbiota to the central nervous system, the gut‐brain axis is comprised of the immune and neuroendocrine systems, hypothalamic–pituitary–adrenal axis, and metabolic pathways [54]. A growing body of preclinical [20] and clinical [55, 56, 57] evidence suggests that anxiety, depression, fatigue, and pain arise from chemotherapy‐induced disruption of the gut‐brain axis. Likewise, our prior research identified perturbations in this pathway that were associated with increased nausea [27], anxiety [24], morning and evening fatigue [26], sleep disturbance [30], and shortness of breath [31].

### 4.2 Helper T (Th)17 Cell Differentiation

Th17 cells, found primarily in the lamina propria of mucosal tissues, play a central role in mucosal barrier homeostasis [58]. Following microbial activation of antigen‐presenting cells, pro‐inflammatory cytokines (i.e., IL‐1B, IL‐6, IL‐23, transforming growth factor beta) bind to naïve cluster of differentiation (CD) 4+ T cells and trigger Th17 cell differentiation [58]. These Th17 cells induce the production of IL‐17A, IL‐17F, IL‐21, and IL‐22 that increase the inflammatory responses of neutrophils and epithelial cells. Given that the mucosal tissues of the gastrointestinal tract are exposed to commensal bacteria routinely, this microbial inflammatory response is held in check by other cytokines (e.g., IL‐4, IL‐27) that suppress Th17 cell differentiation [59].

Of note, gastrointestinal dysbiosis may alter this balance. For example, in one preclinical study [60], compared to controls, mice that received cyclophosphamide exhibited increased permeability of the small intestine; translocation of pathogens to secondary lymph organs; and alterations to microbial composition and Th17 differentiation. In addition, gastrointestinal dysbiosis and alterations to Th17 differentiation are implicated in various chronic conditions, including major depressive disorder [61] and inflammatory bowel disease (IBD) [59]. In our previous analyses, this pathway was associated with anxiety [24], morning and evening fatigue [26], sleep disturbance [30], shortness of breath [31], and pain [29].

### 4.3 Nucleotide‐Binding Oligomerization Domain (NOD)‐like Receptor Signaling Pathway

NOD‐like receptors are pattern recognition receptors that activate the innate immune response [62] and engage in intestinal host‐microbiome homeostasis [63]. In response to stress, epithelial cell damage, and/or damage‐ or pathogen‐associated molecular patterns, NOD‐like receptors activate inflammasomes, as well as the nuclear factor kappa B (NF‐kappa B) and mitogen‐activated protein kinase (MAPK) signaling pathways that coordinate the production of various pro‐inflammatory cytokines [64]. While this inflammatory response is essential to address an infectious insult, promote tissue repair, and support epithelial stem cell survival [63], dysregulation of this pathway is implicated in a variety of acute (e.g., coronavirus disease 2019 [64]) and chronic (e.g., major depressive disorder [65], IBD [62], HIV [66]) conditions. Of note, all of these conditions are characterized by a high symptom burden (e.g., patients living with HIV infection report an average of 9.7 concurrent symptoms [67]). Furthermore, associations between NOD‐like receptor dysregulation and various common symptoms were reported in our previous studies of cognitive impairment [25], morning and evening fatigue [26], depression [unpublished], sleep disturbance [30], shortness of breath [31], and pain [29].

### 4.4 Phosphatidylinositol 3‐Kinase Protein Kinase B (PI3K‐Akt) Signaling Pathway

In addition to its role in DNA repair, metabolism, and cell proliferation, growth, and survival, the PI3K‐Akt signaling pathway is central to the inflammatory response [68]. Upon activation by various stimuli, including antigens, cytokines, chemokines, pathogen‐associated molecular patterns, and growth factors, PI3K catalyzes the production of phosphatidylinositol‐3, 4, 5‐triphosphate which in turn activates two kinases, namely Akt and 3‐phosphoinositide‐dependent protein kinase‐1 [68]. These kinases mediate various cellular processes and inflammation through phosphorylation of various downstream substrates and interactions with multiple pathways (e.g., NF‐kappa B, MAPK) [68]. PI3K‐Akt signaling is negatively regulated through reversal of Akt activation [69].

Dysregulation of this pathway leads to increased oxidative stress and peripheral and central inflammation that are associated with various chronic conditions (e.g., depression [70], chronic obstructive pulmonary disease [69]) and cancer [71]. In addition, our prior research identified associations between this pathway and anxiety [24], evening fatigue [26], depression [unpublished], nausea [28], and shortness of breath [31].

### 4.5 NF‐Kappa B Signaling Pathway

Chemotherapy‐induced damage to epithelial cells of the gastrointestinal tract triggers the production of various pro‐inflammatory cytokines (e.g., IL‐1B, TNF) and reactive oxygen species and activates NF‐kappa B signaling [51]. In addition to its role in supporting adaptive immunity through B and T cell survival and differentiation [72, 73], NF‐kappa B is a transcription factor that activates the inflammatory response by upregulating the production of various cytokines, chemokines, and vascular adhesion molecules. Dysregulation of NF‐kappa B signaling is associated with chronic stress [74] as well as with various inflammatory (e.g., IBD [72]) and neurodegenerative (e.g., Alzheimer's disease [73]) conditions. Our previous research found associations between NF‐kappa B pathway dysregulation and morning fatigue [26], nausea [27], and shortness of breath [31]. In addition, in our previous work that evaluated for associations between expression‐associated cytosine‐phosphate‐guanine (CpG) loci and symptom clusters, findings suggested that increased expression of two genes in this pathway (i.e., cluster of differentiation 40, lymphotoxin beta) was associated with the psychological [75] and gastrointestinal [76] symptom clusters.

### 4.6 MAPK Signaling Pathway

Initiated by multiple stimuli including cytokines (e.g., IL‐1B, TNF), lipopolysaccharides, oxidative stress, and growth factors [77], MAPK signaling plays a central role in cellular proliferation, differentiation, metabolism, migration, survival, and inflammation [78]. In addition to our prior reports of associations with anxiety [24], cognitive impairment [25], morning fatigue [26], nausea [27], shortness of breath [31], and pain [29], dysregulation of MAPK is associated with major depressive disorder [77] and other chronic conditions (e.g., Alzheimer's disease [78]).

### 4.7 Antigen Processing and Presentation, Endocytosis, and Phagosome

In order for T cells to recognize and destroy bacteria and viruses, antigen processing and presentation, endocytosis, and phagocytosis, are needed. Antigen processing begins with the proteolysis of bacterial or viral antigens into peptides [79]. Through one pathway, these peptides are associated with major histocompatibility complex (MHC) I molecules for recognition by CD8 T cells [80]. In another pathway, endocytosis or phagocytosis occurs and these antigens are degraded into peptides via lysosomal proteolysis [79]. Then, these peptides are associated with MHC II molecules for recognition by CD4 T cells or with MHC I molecules for recognition by CD8 T cells.

These pathways play important roles in maintaining intestinal homeostasis [81]. However, the administration of chemotherapy can disrupt the intestinal barrier and perturb homeostasis [51]. In our prior work [24, 25, 26, 27, 28, 29, 30, 31], perturbations in antigen processing, endocytosis, and/or phagosome signaling were associated with anxiety [24], cognitive impairment [25], depression [unpublished], morning and evening fatigue [26], nausea [27, 28], sleep disturbance [30], shortness of breath [31], and pain [29].

### 4.8 Cytokine–Cytokine Receptor Interaction

Cytokines are essential signaling molecules of the immune system that activate or are the end result of several inflammatory pathways. The cytokine–cytokine receptor interaction pathway catalogs the complex network of interactions among various types of cytokines and their receptor(s). While cytokines play an essential role in responding to infections, elevated levels of pro‐inflammatory cytokines were identified in previous studies of sickness behavior [14, 15, 16] and stress [74]. In addition, a large body of evidence suggests that elevated levels of circulating cytokines are associated with more severe symptoms in patients with cancer [82]. Of note, perturbations in this pathway were found in our studies of anxiety [24], cognitive impairment [25], depression [unpublished], morning and evening fatigue [26], nausea [27, 28], sleep disturbance [30], shortness of breath [31], and pain [29].

### 4.9 Common Pathways Across Symptom Burden and Single Symptoms

As shown in Table 5, of the 10 pathways that were significantly perturbed between patients with Low and High symptom burden, three or more were identified in our previous PIAs of single sickness behavior symptoms (i.e., anxiety [24], depression [unpublished], cognitive impairment [25], morning and evening fatigue [26], nausea [27, 28], pain [29], sleep disturbance [30], shortness of breath [31]). Of note, none of these 10 pathways was distinct to the symptom burden PIA. These findings suggest that the processes that underlie symptom burden may be the result of additive or synergistic effects among the biological pathways associated with single symptoms.

The cytokine–cytokine receptor interaction and endocytosis pathways were common to all nine of the single symptom analyses and the current symptom burden analysis. Several lines of evidence support the finding that the cytokine–cytokine receptor interaction pathway is a universal pathway. First, the inflammatory effects of the tumor [83], as well as the elevated levels of circulating cytokines pre‐ and post‐chemotherapy [84], are well documented and previous research demonstrated associations between cytokines and various symptoms and symptom clusters [85, 86, 87, 88, 89]. In addition, given the primary role of cytokines as mediators of immune and inflammatory processes [90], dysregulation of the cytokine–cytokine receptor interaction pathway may directly or indirectly impact all of the other pathways that were identified. Of note, all of the inflammatory cytokine genes (i.e., *Cxcl1*, *Il6*, *Il1b*, *Tnf*) that were associated with chemotherapy‐induced sickness behavior in preclinical studies [17, 18, 19, 20, 21] are found in this pathway [47]. Given these findings, a comprehensive evaluation of associations between the gene and protein expression of all of the cytokines in this pathway and single sickness behavior symptoms, as well as higher symptom burden, is warranted.

Endocytosis plays a vital role in regulating inflammation in intestinal tissue [81] and neuroinflammation in the brain [91]. It is plausible that disruption to endocytosis in these regions may result in higher symptom burden and sickness behavior symptoms through activation of the gut‐brain axis and/or neuroinflammation. For example, while clathrin‐mediated endocytosis traffics cytokines across the blood‐brain barrier, disruption to this normal process leads to increased cytokine production (e.g., IL‐1B, TNF), blood‐brain barrier disruption, and neuroinflammation [91]. Importantly, perturbations in clathrin‐mediated endocytosis and impairment of the blood‐brain barrier were associated with chemotherapy‐induced cognitive impairment [91], sleep impairment [92], neurological diseases [93], and mood disorders [94]. Interactions between this pathway and the cytokine–cytokine receptor interaction pathway warrant evaluation for their contribution to symptom burden and single sickness behavior symptoms.

### 4.10 Limitations

Several limitations warrant consideration. First, given the study's cross‐sectional design, an evaluation of how inflammatory pathway perturbations change throughout the treatment trajectory is warranted. Second, given that this analysis evaluated patients receiving chemotherapy, future research needs to determine if similar symptom cutpoints and biological pathways are found during other forms of cancer treatment (e.g., radiation therapy, immunotherapy, surgery) and into survivorship. Third, our sample was largely white, well‐educated, and limited to four cancer types. Therefore, our findings may not generalize to all patients with cancer. Fourth, given that various methods can be used to determine symptom burden [95, 96], future pathway analyses are warranted using different phenotypic approaches (e.g., composite scores, symptom burden profiles determined using latent variable modeling [32]).

Furthermore, given that our prior studies of single symptoms used cutoff scores or latent variable modeling of symptom occurrence [27, 28, 31] or severity [24, 25, 26, 29, 30] to determine the symptom phenotypes as well as different thresholds for statistical significance, the findings reported in Table 5 warrant replication. Of note, the Bonferroni method to control the family‐wise error rate is highly conservative and may have led to rejections of true associations in our analyses of cognitive impairment [25] and nausea [27, 28]. In contrast, FDR correction using the Benjamini–Hochberg method overcomes this limitation [49]. Despite these limitations, the strength of our findings warrants confirmation in independent samples.

## 5 Conclusions

This study is the first to identify differentially perturbed immune and inflammatory signaling pathways associated with symptom burden in oncology patients receiving chemotherapy. Notably, three or more of these pathways were identified in our previous studies of common sickness behavior symptoms (i.e., anxiety [24], depression [unpublished], cognitive impairment [25], morning and evening fatigue [26], pain [29], sleep disturbance [30], nausea [27, 28], shortness of breath [31]). Of note, the cytokine–cytokine receptor interaction and endocytosis pathways were identified across all of the studies of individual symptoms and in the current study of symptom burden. While elevated levels of circulating cytokines were associated with increased severity of individual symptoms (e.g., pain, fatigue) [82], future research needs to evaluate for associations between expression levels of the specific cytokine genes within this pathway and higher symptom burden and the severity of individual symptoms. The development of interventions that target one or more of these pathways has the potential to decrease overall symptom burden, as well as the occurrence and/or severity of symptoms associated with sickness behavior.

## Author Contributions

**Carolyn S. Harris:** conceptualization, writing – original draft, writing – review and editing, formal analysis. **Kord M. Kober:** writing – review and editing, conceptualization, methodology, formal analysis, investigation, supervision. **Joosun Shin:** writing – review and editing, formal analysis, conceptualization. **Lisa Morse:** conceptualization, writing – review and editing. **Kate R. Oppegaard:** conceptualization, writing – review and editing, formal analysis. **Steven Paul:** formal analysis, writing – review and editing. **Marilyn J. Hammer:** conceptualization, writing – review and editing. **Jon D. Levine:** conceptualization, writing – review and editing. **Yvette P. Conley:** supervision, writing – review and editing, conceptualization. **Christine A. Miaskowski:** writing – original draft, conceptualization, methodology, writing – review and editing, funding acquisition, resources, supervision, project administration, formal analysis, investigation.

## Disclosure

The authors have nothing to report.

## Ethics Statement

This study was approved by the Committee on Human Research at the University of California, San Francisco and by the Institutional Review Board at each of the study sites.

## Consent

Voluntary, written, informed consent was obtained from all patients.

## Conflicts of Interest

The authors declare no conflicts of interest.

## Supporting information


**Figure S1:** cam471328‐sup‐0001‐FigureS1.pdf.

**Table S1:** cam471328‐sup‐0002‐TableS1.xlsx.

## Data Availability

Data are available from Dr. Miaskowski following the completion of a data sharing agreement with the University of California, San Francisco.
