# Supplementary material for: Cytokine–Cytokine Receptor Interaction and Endocytosis are Common Pathways for Symptom Burden and Sickness Behavior Symptoms in Oncology Patients Undergoing Chemotherapy
Source: Cancer Med. 2025 Nov 11;14(21):e71328. doi: 10.1002/cam4.71328 (PMC12604674; doi:10.1002/cam4.71328)
Supplement: Supplementary file 1 — Figure S1: cam471328‐sup‐0001‐FigureS1.pdf. [file CAM4-14-e71328-s002.pdf]

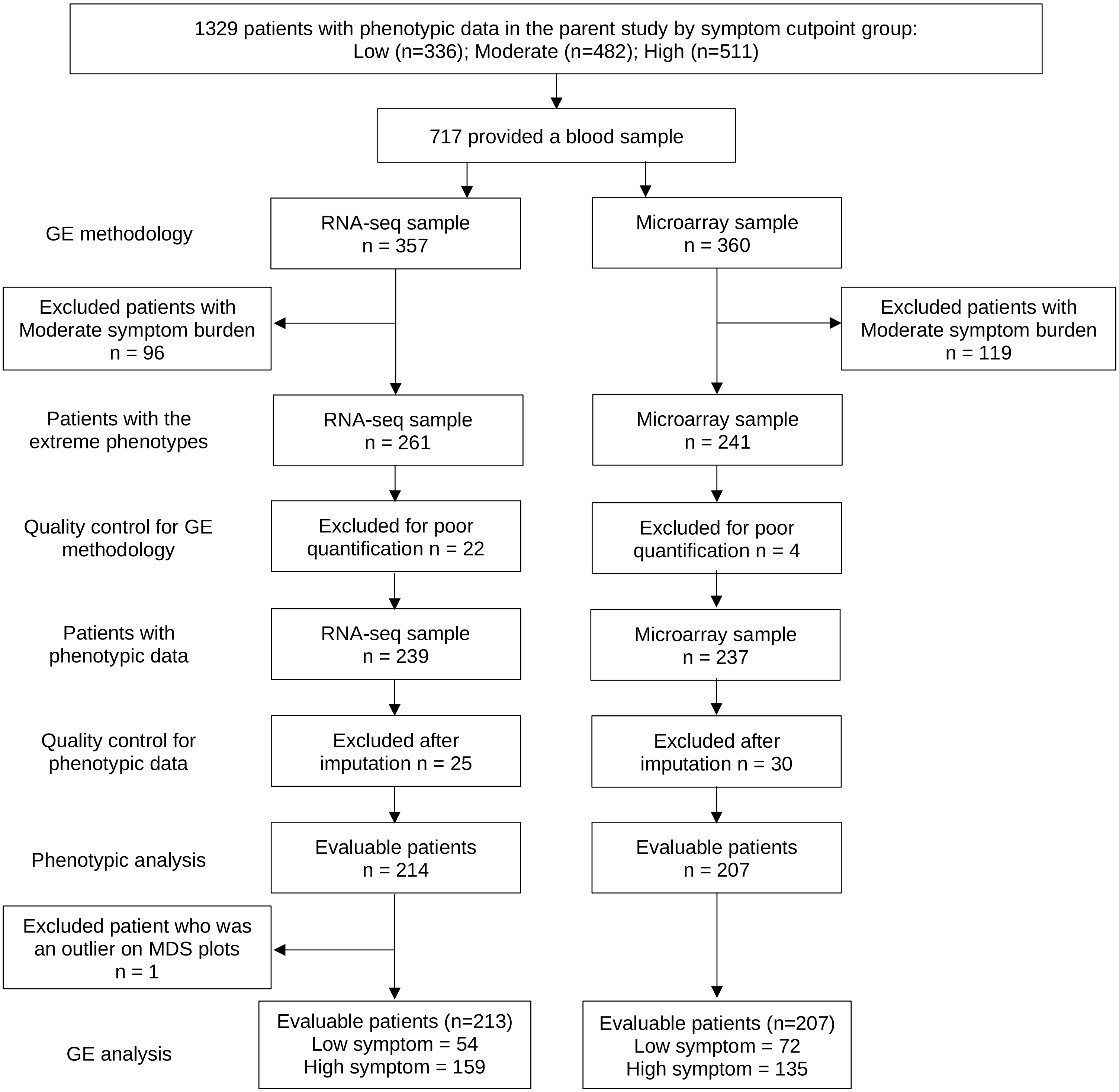

Supplementary Figure 1: Flow diagram of the number of patients available for the phenotypic and gene expression analyses that evaluated for perturbed immune or inflammatory pathways between the Low and High symptom burden groups.

Abbreviations: GE = gene expression; MDS = multidimensional scaling; RNA-seq = ribonucleic acid sequencing
